# Histological Evaluation of Gingival and Intrabony Periodontal Defects Treated with Platelet-rich Fibrin Using Different Protocols: A Canine Study

**DOI:** 10.3290/j.ohpd.b2182985

**Published:** 2021-10-22

**Authors:** Yoshinori Shirakata, Kotaro Sena, Toshiaki Nakamura, Yukiya Shinohara, Takatomo Imafuji, Fumiaki Setoguchi, Kazuyuki Noguchi, Tomoyuki Kawase, Richard J. Miron

**Affiliations:** a Associate Professor, Department of Periodontology, Kagoshima University Graduate School of Medical and Dental Sciences, Kagoshima, Japan. Conceptualisation, methodology, validation, investigation, original draft preparation, visualisation, read and agreed to the submitted version of the manuscript.; b Assistant Professor, Department of Periodontology, Kagoshima University Graduate School of Medical and Dental Sciences, Kagoshima, Japan. Formal analysis, investigation, visualisation, project administration, read and agreed to the submitted version of the manuscript.; c Lecturer, Department of Periodontology, Kagoshima University Graduate School of Medical and Dental Sciences, Kagoshima, Japan. Methodology, investigation, data curation, review and editing, read and agreed to the submitted version of the manuscript.; d Dentist, Department of Periodontology, Kagoshima University Graduate School of Medical and Dental Sciences, Kagoshima, Japan. Investigation, read and agreed to the submitted version of the manuscript.; e Dentist, Department of Periodontology, Kagoshima University Graduate School of Medical and Dental Sciences, Kagoshima, Japan. Investigation, read and agreed to the submitted version of the manuscript.; f Professor, Department of Periodontology, Kagoshima University Graduate School of Medical and Dental Sciences, Kagoshima, Japan. Review and editing, supervision, read and agreed to the submitted version of the manuscript.; g Associate Professor, Division of Oral Bioengineering, Graduate School of Medical and Dental Sciences, Niigata University, Niigata, Japan. Read and agreed to the submitted version of the manuscript.; h Adjunctive Professor, Department of Periodontology, University of Bern, Bern, Switzerland. Conceptualisation, methodology, validation, investigation, original draft preparation, project administration, funding acquisition, read and agreed to the submitted version of the manuscript.

**Keywords:** animal experiment, intrabony defect, gingival recession defect, periodontal wound healing/regeneration, platelet-rich fibrin

## Abstract

**Purpose:**

To histologically compare the effects of platelet-rich fibrin (PRF) produced using different protocols on periodontal wound healing/regeneration in periodontal defects in dogs.

**Materials and Methods:**

Dehiscence-type gingival recession and two-wall intrabony defects were created bilaterally in the maxillary canines and mandibular premolars, respectively, in four beagle dogs. The recession defects were randomly treated with coronally advanced flap (CAF) alone, CAF and PRF produced via fixed-angle centrifugation (F-PRF; Leukocyte and PRF (L-PRF) protocol) or CAF and PRF produced via horizontal centrifugation (H-PRF). After 2 weeks, the two-wall intrabony defects were randomly treated as follows: open flap debridement (OFD), OFD + F-PRF, OFD + H-PRF and OFD + heated albumin with PRF using bio-heat technologies (Alb-PRF). Eight weeks after the 2nd reconstructive surgery, the animals were euthanised for histological evaluation.

**Results:**

In the PRF-applied defects, new bone and new cementum formation occurred to varying degrees regardless of the protocols used to produce PRF. Particularly in the two-wall intrabony defects, new bone formation extended from the host bone toward the coronal region of the defects in the H-PRF applied sites compared with those in the OFD, F-PRF and Alb-PRF groups, and the H-PRF group showed the greatest amount of newly formed cementum.

**Conclusion:**

PRF induced periodontal regeneration in gingival recession and two-wall intrabony defects in dogs. Further studies are needed to determine the optimal protocol for obtaining predictable periodontal regeneration in periodontal defects in humans.

A number of techniques, biomaterials and growth factors have been utilised in modern dentistry to speed the regeneration of either hard or soft tissues.^29-31,35^ While a great deal of recent advancement has been made with respect to using biologics (i.e. recombinant human proteins) as key mediators of tissue regeneration, some disadvantages have also been reported, including their high supra-physiological doses leading to potential inflammation as well as high costs associated with their use.^2,4,33^ Nevertheless, the use of growth factors such as recombinant human platelet derived growth factor (rhPDGF) and bone morphogenetic protein-2 (rhBMP2) has been shown to positively increase tissue formation across a wide variety of clinical procedures in both dentistry and medicine.

The use of autologous platelet concentrates have also gained tremendous momentum in recent years as a modality to stimulate tissue regeneration.^5,6,31^ Platelet rich plasma (PRP) was a first-generation platelet concentrate with use of anti-coagulants that favoured up to a 6–8 fold increase in platelet concentrations.^19-21^ Early experiments revealed the ability for several key growth factors found in blood including PDGF, transforming growth factor-β1 (TGF-β1), and vascular endothelial growth factor (VEGF) to be significantly expressed in higher levels when compared to whole blood favouring the modulation of tissue repair and wound healing.^1,9,18,24,32,34^ Despite its widespread use, one of the main reported drawbacks of PRP includes its use of anti-coagulants, an event that interferes with the natural wound healing process.^8,40^

Recently, platelet rich fibrin (PRF) was proposed as a second-generation platelet concentrate prepared from peripheral blood without the use of anti-coagulants.^7,10^ PRF involves the formation of a fibrin clot following centrifugation and may be utilised as a regenerative fibrin scaffold with a host concentration of platelets and leukocytes and autologous growth factors.^7,10^ More recently, it was demonstrated via a novel quantification method and histological assessment that PRF produced via horizontal centrifugation (H-PRF) led to a greater yield and concentration of platelets and leukocytes when compared to PRF produced via conventional fixed-angle centrifuges (F-PRF).^12,25^ This represented a marked H-PRF ability to greatly concentrate cells found within H-PRF since standard leukocyte and PRF (L-PRF) protocol produced via fixed-angle devices primarily accumulate cells on the back distal surfaces of PRF tubes. On the other hand, cells are typically driven along the back wall of centrifugation tubes at high g-forces with relative difficult to separate properly according to their cell density during the spin cycles during F-PRF production.

One of the main potential limitations of PRF has been its short in vivo turnover rate. Thus, the heat-compression technique with PRF membranes was introduced by Kawase et al.^15^ Furthermore, it was recently demonstrated that heated albumin with PRF utilizing the Bio-Heat technology (Alb-PRF) extended the working properties of PRF from a typical 2–3 week period to 4–6 months^13,14^ with harder texture compared to F-PRF and H-PRF. These findings led to the hypothesis that H-PRF membrane may be capable of inducing more favorable periodontal wound healing/regeneration compared to F-PRF membrane and Alb-PRF plays biological scaffold/carrier particularly in intrabony defects rather than in gingival recession defects with thin marginal soft and hard tissues. Since creation and maintenance of space for periodontal tissue regeneration is expected more in intrabony defects compared to gingival recession defects. To date however, no data exists comparing PRF clots produced via different methods have been utilised to assess the regeneration of periodontal defects histologically. Therefore the purpose of this study was to histologically compare the effects of PRF produced by different protocol (i.e. F-PRF, H-PRF, Alb-PRF) on periodontal wound healing/regeneration in periodontal defects in dogs.

## Materials and Methods

### Animals

Four healthy male beagle dogs, 19 to 23 months of age and weighing 10.50 to 14.71 kg, were chosen in this study. The animals were housed and monitored daily for the duration of the study in the Animal Experimentation Facility Shin Nippon Biomedical Laboratories, Kagoshima, Japan. They were kept in individual cages at 20–26°C, relative humidity of 30–70%, and a 12-hour light/dark cycle. Approximately 300 g of solid food (NVE-10, Nippon Pet Food; Tokyo, Japan) was provided to each animal daily and water was available ad libitum. All procedures during the in-life phase were approved by the ethics committee of the Animal Research Center of Kagoshima University, Japan (Approval No. D19028). This study conformed to the ARRIVE guidelines for preclinical animal studies.

### Surgical Protocol

All surgical procedures were performed under general and local anesthesia using aseptic routines by a single experienced operator (Yo.S.). Before surgical procedures, analgesics (buprenorphine hydrochloride, 0.1 ml/kg; Leptan, Otsuka Pharmaceutical; Tokyo, Japan) and antibiotics (penicillin G procaine and dihydrostreptomycin sulfate aqueous suspension for injection, 0.05 ml/kg; Mycillin Sol Meiji for veterinary use, Meiji Seika Pharma; Tokyo, Japan) were administered intramuscularly. General anesthesia was achieved with a combination of pentobarbital sodium (Somnopentyl, 0.2 ml/kg IV, Kyoritsu Seiyaku; Tokyo, Japan) and medetomidine hydrochloride (Domitor, 0.08 ml/kg IM, Orion; Espoo, Finland) at a level to maintain spontaneous breathing. Local anesthesia was achieved with lidocaine HCl/epinephrine (2%, 1:80,000; Xylocaine; Fujisawa; Osaka, Japan). Prior to the following reconstructive surgeries, dehiscence-type gingival recession defects (5 mm wide and 7 mm deep) were surgically created bilaterally in the maxillary canines as previously reported.^38,39^ The bilateral mandibular third premolars and first molars were carefully extracted to provide enough space for creation of defects and for flap management to allow wound closure with primary intention healing.

### Preparation of PRF and Defect Management

Immediately before reconstructive surgeries, blood was taken from each animal from the external jugular vein (40 ml) without addition of anticoagulant. For F-PRF preparation, 2 tubes of 10 ml of whole blood without anticoagulants were drawn in silica-coated plastic red tubes (Intra-Lock; Boca Raton, FL, USA) and centrifuged at 2700 rpm (~400 g at the RCF-clot and ~700 g at the RCF-max) for 12 min on a fixed-angled centrifuge machine (Intra-Spin system, Intra-Lock). For H-PRF preparation, horizontal centrifugation was carried out using 2 tubes of 10 ml of blood in glass tubes (Bio-PRF; Venice, FL, USA) using at 700 RCF for 8 minute protocol (Bio-PRF). For preparation of Alb-PRF (or extended-PRF; e-PRF), blood samples were collected in plastic PET tubes without any additives. First, two tubes were centrifuged for 700 RCF for minutes in the horizontal rotor centrifuge (Bio-PRF), and the protocol for H-PRF was applied to obtain the liquid phase (serum + platelet-poor plasma (PPP) + platelet-rich fraction). Approximately 2 ml of the upper PPP portion of plasma was collected using an 18G needle in a 3 ml syringe while the other blood portions (portion rich in cells, and red blood cells) was placed in the Bio-Cool device (Bio-PRF). The syringes containing PPP were then inserted into a heating device for human serum denaturation of proteins (Bio-Heat, Bio-PRF). After heating for 10 min at 75°C, the syringes were then rapidly cooled using the Bio-Cool device for 1–2 min according to the manufacturer’s recommendation. Only thereafter, a 1 ml portion of the rich cell layer from the buffy coat was collected and mixed together with the heated PPP layer (denatured albumin gel) to create Alb-PRF.

### Gingival Recession Defect (1st Reconstructive Surgery in the Maxilla)

The created defects in the maxillary canines were exposed for a period of 8 weeks to plaque accumulation. After 8 weeks of plaque accumulation, a regimen of plaque control using a 2% solution of chlorhexidine gluconate (5% HIBITANE, 25 ml of a 2% solution; Sumitomo Dainippon Pharma; Osaka, Japan) was instituted for 2 weeks prior to the reconstructive surgery.

**Fig 1:**
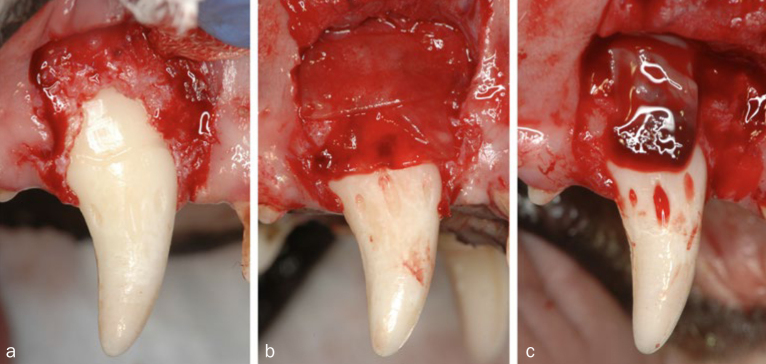
Clinical overview of the treated dehiscence defects. a. Defect on the root after debridement (CAF alone: CAF group). b. F-PRF was placed onto the denuded root surface (F-PRF group). c. H-PRF was placed onto the denuded root surface (H-PRF group).

Full-thickness flaps were then raised, and the root surfaces were scaled to completely remove the biofilm and the residual inflamed granulation tissue. Reference notches were made using a #1 round bur on the root surface at the base of the defects, at the level of the cementoenamel junction (CEJ) and on the crown surface to indicate the precise center plane of the dehiscence defects and to aid in optimal histological processing. Bilateral defects were randomly assigned to receive coronally advanced flap (CAF) alone (Fig 1a), CAF with F-PRF placement (Fig 1b) and CAF with H-PRF placement (Fig 1c). A periosteal releasing incision was made to allow tension-free coronal advancement of the flaps and sutured using non-resorbable monofilament suture material (Gore-Tex Suture CV-6. W.L.; Flagstaff, AZ, USA) with sling and interrupted sutures.

### Two-wall Intrabony Defect (2nd Reconstructive Surgery in the Mandible)

**Fig 2:**
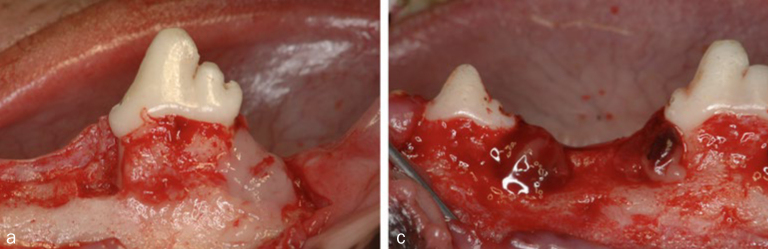
Clinical photographs showing the surgically created and treated two-wall intrabony defects. a. Left: open flap debridement (OFD); right: Alb-PRF application. b. Left: H-PRF was placed into the defect; right: F-PRF placement.

After a 10-week extraction healing interval, two-wall intrabony defects (5 mm wide and 5 mm deep) were prepared bilaterally at the mesial and distal aspects of the mandibular fourth premolars (P4) and at the distal aspect of the mandibular second premolars (P2) (six defects per dog). Following elevation of the mucoperiosteal flap, defects were created by using fissure burs with a sterile saline coolant (Fig 2). Cementum was removed using Gracey curettes and a chisel. Reference notches were made with a #1 round bur on the root surface at the base of the defects, at the CEJ, and on the crown surface, to indicate the precise center plane of the intrabony defects and allow for optimal histomorphometric analysis. The 24 bilateral mandibular two-wall intrabony defects randomly received one of the following treatments after surgical debridement: applications of F-PRF, H-PRF and Alb-PRF, and open flap debridement (OFD) only as a surgical control (Fig 2a).

A periosteal releasing incision was made to allow coronal displacement of the flap, followed by horizontal mattress and interrupted suturing (Gore-Tex CV-6 Suture) slightly coronal to the CEJ.

### Postsurgical Protocol

The animals were fed a soft diet for 2 weeks postoperatively, and then the sutures were removed. Ketoprofen for analgesia (Capisten IM 50 mg, 2 mg/kg, 0.1 ml/kg; Kissei Pharmaceutical; Matsumoto, Japan) and an antibiotic (Mycillin Sol) were administered daily for 2 days. Plaque control was maintained with routine (3 times a week) flushing of the oral cavity with 2% chlorhexidine gluconate solution after the reconstructive surgeries.

### Histologic Preparation

Eight weeks after the 2nd reconstructive surgery, the animals were euthanised with an overdose of sodium thiopental. All defects were then resected, together with the surrounding soft and hard tissues. The tissue blocks were fixed in 10% buffered formalin, trimmed, and rinsed in phosphate-buffered saline. The samples in maxilla were dehydrated and embedded in polyester resin. The resin blocks were cut bucco-lingually to a thickness of 100 to 150 μm with a low-speed diamond saw. Slides were ground and polished to a final thickness of 35–45 μm using a microgrinding system with non-adhesive abrasive discs and stained with toluidine blue. The samples in mandible were decalcified in KalkitoxTM (Wako Pure Chemical Industries; Osaka, Japan), dehydrated, and embedded in paraffin. Serial 6-µm-thick sections were then prepared along the mesiodistal plane and were stained with hematoxylin and eosin. The reason for using paraffin histology in addition to undecalcified ground sections was to observe more clearly cell morphology including the orientation/density of the collagen fibers and to keep some samples available for potential future immunohistochemical evaluation.

### Histomorphometric Analysis

All specimens were analysed under a light microscope (BX51, Olympus; Tokyo, Japan) equipped with a computerised image system (WinROOF2015, Mitani; Tokyo, Japan).

### Gingival Recession Defects

For the histomorphometric analysis, 2 sections were selected from the most central area of each gingival recession defect in the maxilla, identified by the coronal and apical notches on the root and the reference notch on the crown. The following parameters were measured by a single experienced blinded examiner (T.N.): 1. defect height: distance between the apical extent of root planing and the CEJ; 2. junctional epithelium length: distance between the apical extension of the junctional epithelium and the CEJ; 3. connective tissue adhesion (without cementum): distance between apical extent of the junctional epithelium and the coronal extent of the newly formed cementum; 4. new bone length: distance between the apical extent of root planing and the coronal extent of newly formed alveolar bone along the root surface; 5. new cementum length: distance between apical extent of root planing and coronal extent of newly formed cementum on the denuded root surface. 6. soft tissue height (STH): distance between apical extent of root planing and gingival margin; 7. soft tissue thickness (STT): distance from the buccal outmost gingival/mucosal surface to the tooth surface at three different levels, STT-1: at the top of the coronal notch (CEJ), STT-2: at the middle between the coronal and apical notches, and STT-3: at the base of the apical notch (defect); 8. gingival recession (GR): distance from the gingival margin to the coronal notch (CEJ). The mean value of each histomorphometric parameter was then calculated for each site.

### Two-wall Intrabony Defects

For the histomorphometric analysis, 3 sections approximately 90 µm apart were selected from the most central area of each two-wall defect in the mandible, identified by the length of the root canal and the reference notches. Instead of the measurements of STH, STT and GR, the following parameter was also measured by the same examiner: 9. new bone area: newly formed trabecular bone within a template (5x5 mm) that served as a standardised proxy for the defect site. The template was aligned parallel to the root surface interfacing the apical extension of the root planing.^17^

### Statistical Analysis

No statistical analysis was performed due to limited number of samples in maxilla. The means and standard deviations for each parameter were calculated for each of the four treatment groups (n = 6 defect sites/group) performed in the mandible. The mean scores were used to test for differences among the experimental treatments using one-way ANCOVA and a post-hoc (Bonferroni) test for multiple comparisons. A p-value of < 0.05 was considered statistically significant. All calculations were performed with statistical software (BellCurve for Excel; Social Survey Research Information; Tokyo, Japan).

## Results

### Clinical Observations

Postoperative clinical healing was uneventful at all 32 sites. No visible adverse reactions, including suppuration, abscess formation, or increased tooth mobility, were observed throughout the entire experimental period.

### Descriptive Histology

#### Gingival recession defects

**Fig 3:**
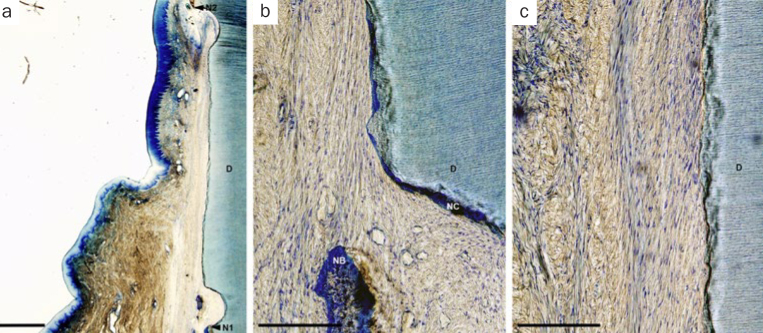
Representative photomicrographs of a gingival recession defect treated by CAF. a. Overview of the defect (scale bar, 1 mm; toluidine blue stain); b. higher magnification of the notch area (scale bar, 200 μm; toluidine blue stain); c. higher magnification of the middle portion of the defect (scale bar, 200 μm; toluidine blue stain). D: root dentin; N1: apical notch; N2: coronal notch (CEJ: cementoenamel junction); NB: new bone; NC: new cementum.

**Fig 4:**
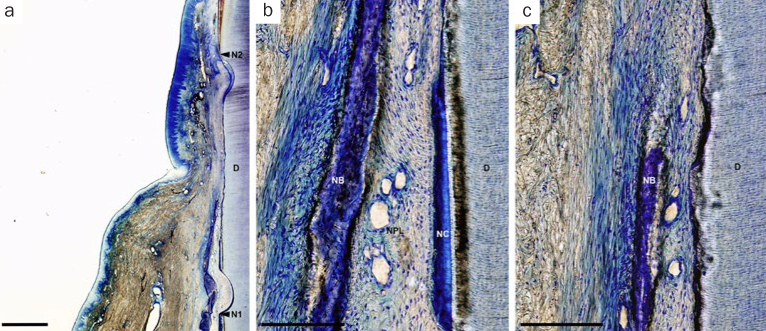
Representative photomicrographs of a gingival recession defect treated by CAF with F-PRF. a. Overview of the defect (scale bar, 1 mm; toluidine blue stain); b. higher magnification of the apical portion of the defect (scale bar, 200 μm; toluidine blue stain); c. higher magnification of the middle portion of the defect (scale bar, 200 μm; toluidine blue stain). D: root dentin; N1: apical notch; N2: coronal notch (CEJ: cementoenamel junction); NB: new bone; NC: new cementum; NPL: new periodontal ligament.

**Fig 5:**
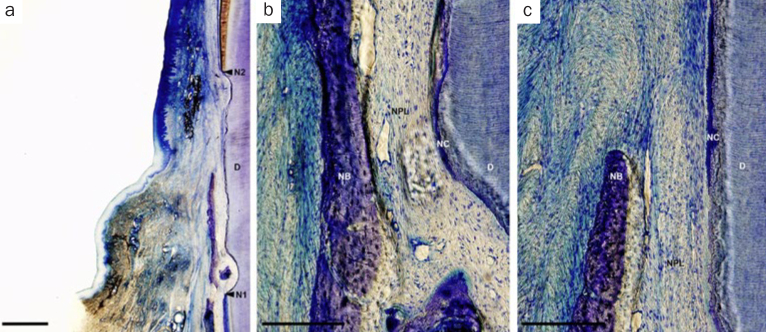
Representative photomicrographs of a gingival recession defect treated by CAF with H-PRF. a. Overview of the defect (scale bar, 1 mm; toluidine blue stain); b. higher magnification of the apical portion of the defect (scale bar, 200 μm; toluidine blue stain); c. higher magnification of the middle portion of the defect (scale bar, 200 μm; toluidine blue stain). D: root dentin; N1: apical notch; N2: coronal notch (CEJ: cementoenamel junction); NB: new bone; NC: new cementum; NPL: new periodontal ligament.

In the CAF group, minimal new bone formation was observed. Broad connective tissue adhesion without cementum was observed and connective tissue fibers were aligned parallel to the root surface (Fig 3). Generally, histological findings in the F-PRF-applied sites were similar to that in the H-PRF-applied sites. In some of the PRF-applied (F-PRF and H-PRF) sites, bone formation was noted extending from apical notches towards the coronal region of the defects. In these defects, dense collagen fibers were seen inserting into the newly formed cementum, oriented oblique to the root surfaces (Figs 4 and 5). However, no apparent periodontal regeneration was observed in one animal regardless of the application of F-PRF or H-PRF.

#### Two-wall intrabony defects

**Fig 6:**
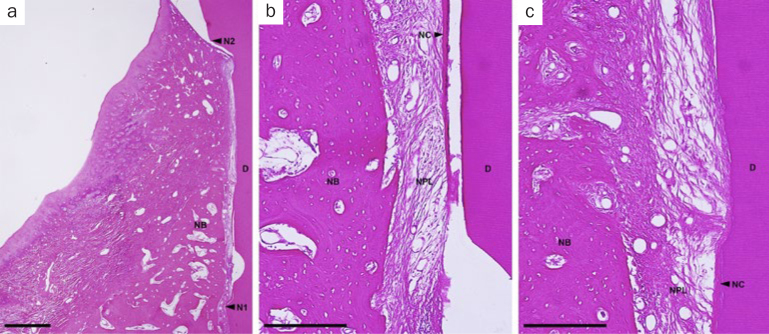
Representative photomicrographs of a two-wall intrabony defect treated by OFD. a. Overview of the defect (scale bar, 1 mm; hematoxylin and eosin stain); b. higher magnification of the apical portion of the defect (scale bar, 200 µm; hematoxylin and eosin stain); c. higher magnification of the portion of the bone crest (scale bar, 200 µm; hematoxylin and eosin stain). D: root dentin; N1: apical notch; N2: coronal notch (CEJ: cementoenamel junction); NB: new bone; NC: new cementum; NPL: new periodontal ligament.

**Fig 7:**
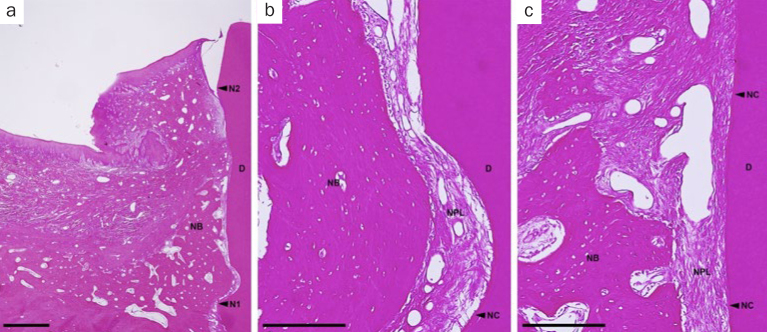
Representative photomicrographs of a two-wall intrabony defect treated by OFD and Alb-PRF. a. Overview of the defect (scale bar, 1 mm; hematoxylin and eosin stain); b. higher magnification of the apical portion of the defect (scale bar, 200 µm; hematoxylin and eosin stain); c. higher magnification of the portion of the bone crest (scale bar, 200 µm; hematoxylin and eosin stain). D: root dentin; N1: apical notch; N2: coronal notch (CEJ: cementoenamel junction); NB: new bone; NC: new cementum; NPL: new periodontal ligament.

**Fig 8:**
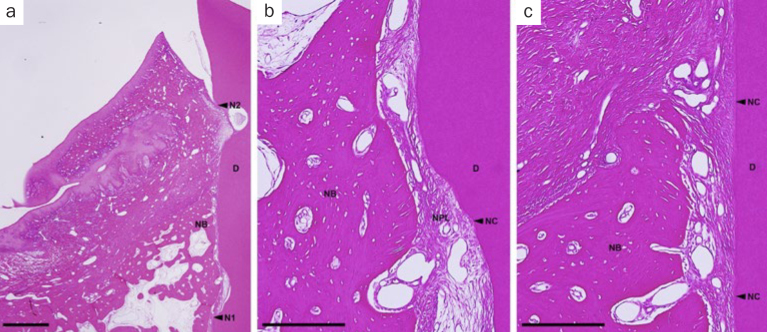
Representative photomicrographs of a two-wall intrabony defect treated by OFD and F-PRF. a. Overview of the defect (scale bar, 1 mm; hematoxylin and eosin stain); b. higher magnification of the apical portion of the defect (scale bar, 200 µm; hematoxylin and eosin stain); c. higher magnification of the portion of the bone crest (scale bar, 200 µm; hematoxylin and eosin stain). D: root dentin; N1: apical notch; N2: coronal notch (CEJ: cementoenamel junction); NB: new bone; NC: new cementum; NPL: new periodontal ligament.

**Fig 9:**
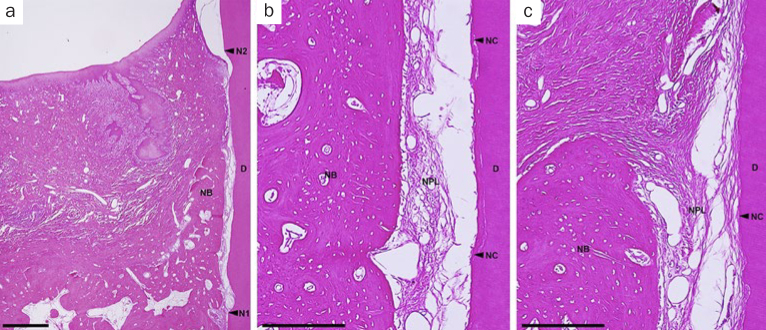
Representative photomicrographs of a two-wall intrabony defect treated by OFD and H-PRF. a. Overview of the defect (scale bar, 1 mm; hematoxylin and eosin stain). b. higher magnification of the apical portion of the defect (scale bar, 200 µm; hematoxylin and eosin stain); c. higher magnification of the portion of the bone crest (scale bar, 200 µm; hematoxylin and eosin stain). D: root dentin; N1: apical notch; N2: coronal notch (CEJ: cemento-enamel junction); NB: new bone; NC: new cementum; NPL: new periodontal ligament.

In the OFD group, spontaneous bone formation occurred to some extent. New cementum formation was restricted below the bone crest in most sites. New cellular cementum was seen, with or without collagen fibers oriented parallel to or detached from the root surfaces (Fig 6). In the PRF-applied groups, new bone formation occurred to varying degrees in all PRF groups. However, new bone formation extended from the host bone toward the coronal region of the defects in the H-PRF applied sites compared with those in the F-PRF and Alb-PRF applied sites (Fig 9). New cellular/acellular cementum, with inserting collagen fibers running perpendicular to the root surfaces, was dominantly observed in all PRF groups when compared to the OFD group (Figs 7 to 9). The highly vascularised and dense new periodontal ligament-like tissue, which was formed between the new cementum and new bone, maintained its width up to the coronal portion in the PRF-applied groups (Figs 7 to 9). Neither root resorption nor ankylosis was observed in any of the defects.

#### Histomorphometric analysis

The results from histomorphometric analysis for the gingival recession defects are shown in Table 1. The histomorphometric parameters (NC, NB, STH, and STT-2) in the PRF groups were greater than those obtained in the CAF group. Particularly, the length of NC in the F-PRF and H-PRF groups was greater compared with that in the CAF group, although without statistically significant differences between the F-PRF and H-PRF groups. The length of GR in the CAF group was greater than that in the PRF-applied groups. The results of histomorphometric analysis for two-wall intrabony defects are shown in Table 2. No statistically significant differences were detected among the groups in any of the measured histomorphometric parameters. Nevertheless, the length of CT (without cementum formation) in the H-PRF group was the smallest, and the H-PRF group showed the greatest amount of newly formed cementum.

**Table 1:** Histomorphometric analysis in gingival recession defects (mean ± SD, n = 4 animals, n = 8 defect sites in total)

Parameter	Treatment group		
	CAF (n = 2)	F-PRF (n = 3)	H-PRF (n = 3)
DH (mm)	6.31 ± 0.61	5.23 ± 0.09	4.76 ± 1.36
JE (mm)	1.43 ± 1.35	2.21 ± 0.81	0.90 ± 0.31
CT (mm)	3.13 ± 4.11	1.59 ± 0.49	2.54 ± 1.15
NC (mm)	0.10 ± 0.15	1.03 ± 1.38	0.89 ± 1.54
NB (mm)	0.24 ± 0.34	0.78 ± 0.42	0.46 ± 0.57
STH (mm)	5.05 ± 3.47	6.61 ± 1.14	6.38 ± 1.87
STT-1 (mm)	0.42 ± 0.59	0.78 ± 0.42	0.48 ± 0.57
STT-2 (mm)	0.62 ± 0.88	1.38 ± 0.60	1.23 ± 0.60
STT-3 (mm)	2.95 ± 0.27	2.19 ± 0.98	2.14 ± 0.88
GR (mm)	1.63 ± 2.31	0.01 ± 0.02	0.33 ± 0.57

CAF: coronally advanced flap; F-PRF: PRF produced via fixed-angle centrifuge; H-PRF: PRF produced via horizontal centrifuge; DH: Defect height; JE: Length of junctional epithelium; CT: connective tissue adhesion without cementum; NC: Length of new cementum formation; NB: Length of new bone formation; STH: soft tissue height; STT-1: soft tissue thickness at the CEJ; STT-2: soft tissue thickness at the middle between the CEJ and apical notch; STT-3: soft tissue thickness at the base of the apical notch; GR: gingival recession.

**Table 2:** Histomorphometric analysis in two-wall intrabony defects (mean ± SD, n = 4 animals, n = 24 defect sites in total)

Parameter	Treatment group			
	OFD (n = 6)	F-PRF (n = 6)	Alb-PRF (n = 6)	H-PRF (n = 6)
DH (mm)	5.04 ± 0.53	4.55 ± 0.38	5.17 ± 0.50	5.10 ± 0.32
JE (mm)	0.95 ± 0.63	0.71 ± 0.44	0.54 ± 0.39	1.04 ± 0.56
CT (mm)	2.38 ± 1.78	1.88 ± 1.18	2.42 ± 1.67	0.96 ± 0.81
NC (mm)	1.41 ± 1.06	1.80 ± 1.11	2.06 ± 1.36	3.09 ± 0.58
NB (mm)	2.06 ± 0.72	1.74 ± 1.00	1.82 ± 0.80	2.16 ± 0.96
NBA (mm^2^)	2.25 ± 1.43	1.78 ± 1.44	2.03 ± 1.75	2.43 ± 1.20

OFD: open flap debridement; F-PRF: PRF produced via fixed-angle centrifuge; Alb-PRF: heated albumin with PRF using bio-heat technologies; H-PRF: PRF produced via horizontal centrifuge; DH: defect height; JE: junctional epithelium length; CT: connective tissue adhesion (without cementum); NC: new cementum length; NA: new attachment length; NB: new bone length; NBA: new bone area (newly formed trabecular bone within a 5x5-mm template).

## Discussion

To the best of our knowledge, the present study is the first preclinical trial to histologically evaluate the effects of PRF produced by different protocols on periodontal wound healing/regeneration in gingival recession and two-wall intrabony defects. The gingival recession defects treated by CAF alone demonstrated minimal new bone and new cementum formation with broad connective tissue adhesion (without cementum). The histological finding is similar to those of previous studies demonstrating limited periodontal regeneration on the root surfaces treated by CAF alone^38,39^ or CAF with connective tissue graft (CTG).^3,22,41^ In the F-PRF and H-PRF groups, new bone formation occurred extending from the apical notches toward the coronal region of the defects in all but one defect. On the other hand, Suaid et al. (2008) demonstrated that a small amount of bone resorption was found in almost all teeth following PRP and CTG application in a similar gingival recession defect model in dogs.^41^ These results indicate that PRF may be better suited for periodontal regeneration when compared to PRP since a longer growth factor release profile was observed as well as lack of any type of chemical additive commonly found in PRP. In addition, new attachment characterised by dense collagen fibers inserting into the newly formed cementum and new bone was observed in the PRF-applied defects. These findings are also in agreement with the histological results obtained in gingival recession defects treated by PDGF,^23^ enamel matrix derivative^22,39^ and hyaluronic acid gel,^38^ and support that F-PRF and H-PRF play important roles as autologous/natural-bioactive agents that promote favorable periodontal regeneration. The amounts of STH and STT2 were also greater in the PRF-applied groups than in the CAF group. The favorable soft tissue healing may be supported by the report that PRF statistically significantly promoted the expression of collagen production-rich genes (COL1A1, COL3A1, and TIMP1) and growth factors (PDGF, TGFβ1, and VEGFA).^16^ These findings may explain the clinical outcomes that the use of PRF in conjunction with CAF statistically significantly improve percentage of relative root coverage when compared with CAF alone.^27^ However, neither F-PRF nor H-PRF promoted periodontal regeneration in an individual animal with very thin (< 1mm) buccal alveolar bone plate and/or limited keratinised mucosa width at the time of reconstructive surgery. Thus, the use of CTG and/or collagen matrix may be preferred over PRF for thin phenotypes to facilitate more predictable periodontal wound healing/regeneration.^27,39^ This is supported by a recent systematic review that concluded that while PRF was additionally favorable for gingival recession coverage, adequate baseline keratinised tissue was needed for its use.^27^

In two-wall intrabony defects, Alb-PRF was also employed to evaluate the potential scaffold effects on periodontal wound healing/regeneration when compared with OFD, F-PRF and H-PRF. New bone formation occurred towards the coronal region of the two-wall intrabony defects in all of the groups without statistically significant differences in NB and NBA measurements. However, previous clinical studies have shown that PRF in conjunction with OFD led to a statistically significant improvement not only in clinical parameters (i.e. probing depth, clinical attachment level) but also radiographic bone fill values, with comparable outcomes to OFD with bone grafts.^28^ Moreover, a recent animal study showed that PRF statistically significantly enhanced the osteoblastic activity of alveolar bone demonstrated by higher expressions of osteocalcin and osteopontin compared with blood clot alone in extraction sockets in dogs.^42^ The discrepancy regarding the effect of PRFs on bone formation in this study may be attributed to the different defect types, or may be due to the acute type of two-wall intrabony defects observed in dogs,^37^ with a turnover rate of bone remodeling approximately four times faster than in humans.^11^

Despite this, all PRF-applied groups demonstrated new cellular/acellular cementum, with inserting collagen fibers running perpendicular to the root surfaces, was dominantly observed when compared to the OFD alone group. These results also indicate that all PRFs contributed to periodontal regeneration to some extents. However, no statistically significant differences in the histomorphometric parameters (i.e. JE, CT and NC) were observed between the PRF-applied groups. These findings suggest that platelet activation is induced in each of the fibrin matrices. Noteworthy, the H-PRF group demonstrated greater amounts of NB, NBA and NC than those in the OFD, F-PRF and Alb-PRF groups and the results may be supported by the fact that horizontal centrifugation lead to up to a 4 times greater cell content when compared to fixed-angle centrifugation.^25^ It was also observed that periodontal regeneration appeared histologically superior in both the F-PRF and H-PRF groups when compared to Alb-PRF. A recent study performed according to ISO 10993 standards found that while F-PRF and H-PRF were fully resorbed within a 2–3 week period with all growth factors being released within this time frame, the Alb-PRF lasted the equivalent of 4–6 months.^14^ It has also been reported that the fast resorption of residual biomaterials may be better desirable during periodontal regeneration to avoid the risk for infection and to increase the amount of regenerated tissues in periodontal defects.^36,43^ Thus, it may be that the longer resorption period of the Alb-PRF is not beneficial to periodontal regeneration when compared to standard PRF protocols and it may be that the use of Alb-PRF is better utilised instead as a barrier membrane with a longer ability to exclude soft tissues when compared to standard PRF. Future studies to characterize and define the recommended clinical uses of Alb-PRF remain needed.

## Conclusion

Within the limits of this preclinical study, it can be concluded that PRF induced periodontal regeneration in gingival recession and two-wall intrabony defects in dogs. The blood cell counts measured (e.g. mean platelet count: 263.0 x 10^3^/μl, erythrocyte count: 7.8 x 10^6^/μl, and leukocyte count: 16.2 x 10^3^/μl) were normal, without large variations between dogs and humans. The final PRF membranes produced in this study were therefore very similar to those found in humans especially in terms of platelet concentrations. One noticeable difference however between the PRF produced in this study compared to humans was the clearer texture of the plasma layer when compared to a random human patient population. The final PRF produced from this animal model was very consistent between animals both in size and shape, whereas based on the authors’ experience, a greater variability exists in human patients. One hypothesis described in the literature^26^ in human studies has been the effects of high cholesterol/fat intake in human samples, often appearing more cloudy in appearance in the plasma layer. Since all animals in this study were between 1.5–2 years of age, eating a well-controlled diet with adequate exercise, it was noted that the produced PRF was more consistent in texture without as much variability noted in a human population. Further studies are nevertheless required to determine the optimal protocol for reducing this variability and obtaining predictable periodontal regeneration for treatment of periodontal defects in humans
